# Noncontiguous Multifocal Spondylodiscitis in 3 Regions of the Spine

**DOI:** 10.1155/2022/2091676

**Published:** 2022-11-26

**Authors:** Fernando González González, Arturo Aguirre Madrid, Dizán Mendoza Pedroza, Abelardo Loya Solis, Fernando Hernández Aragon, Nadia Karina Portillo Ortiz, Edmundo Berumen Nafarrate

**Affiliations:** ^1^Department of Orthopedic Surgery, Christus Muguerza del Parque Hospital, de la Llave St. No. 1419, Office 9, Col. Centro, 31000 Chihuahua, Chihuahua, Mexico; ^2^Medical Specialties, Vice-Rectory of Health Sciences, Universidad de Monterrey, Ignacio Morones Prieto Ave. 4500 W, San Pedro Garza Garcia, Nuevo Leon 66238, Mexico; ^3^Department of Radiology and Imaging, Christus Muguerza del Parque Hospital, Dr. Pedro Leal St. No. 1802, Col. Centro, 31000 Chihuahua, Chihuahua, Mexico; ^4^Department of Pathological Anatomy, Christus Muguerza del Parque Hospital, Dr. Pedro Leal St. No. 1802, Col. Centro, 31000 Chihuahua, Chihuahua, Mexico; ^5^Faculty of Medicine and Biomedical Sciences, University Autonomous of Chihuahua, 31125 Chihuahua, Chihuahua, Mexico

## Abstract

Spondylodiscitis is an uncommon infectious disease of the spine, usually presenting in 1 or 2 contiguous levels, associated with risk factors such as diabetes, intravenous drugs, corticosteroids, and invasive procedures. The most common presentation is pain with nonspecific systemic manifestations. Diagnosis relies on clinical suspicion, laboratories, and imaging studies. Urgent treatment is important due to the high morbid mortality associated with sepsis or a fulminant disease course. We report the case of a 39-year-old female diagnosed with noncontiguous multifocal spondylodiscitis, in the cervical, thoracic, and lumbar spine. The patient initially presented with back pain, inability to walk and severe neurological deficit in the upper and lower extremities, upon diagnosis broad-spectrum antibiotics were initiated. A staged surgical approach was performed in the 3 spine segments. During the 6 month follow-up, the patient presented walking with assistance, with the recovery of strength in the upper and lower extremities.

## 1. Introduction

Spondylodiscitis is an infectious disease characterized by an inflammatory process affecting the vertebral body (spondylitis), intervertebral disc (discitis), and associated paravertebral soft tissue. It is an uncommon pathology, the incidence is thought to be 4-24 per million per year in developing countries, accounting for around 2%-5% of all osteomyelitis. Spinal infections commonly present in one or two contiguous levels, most commonly in the lumbar spine and are related to risk factors such as diabetes, chronic steroid use, intravenous drug use, and invasive surgical procedures. Although several organisms have been associated with spondylodiscitis (bacterial, viral, and fungal), *Staphylococcus aureus* remains the main pathogen, accounting for 20-80% of the cases. The clinical presentation is insidious and nonspecific, with chronic neck or back pain and systemic symptoms, contributing to a prolonged time between onset and diagnosis [1, 2].

Pathogens can colonize the spine mainly through 3 routes: a contiguous infection, direct inoculation, and hematogenous dissemination, the latest being the most common. Understanding the pathophysiology requires knowledge of the vascular anatomy; the aorta forms multiple intercostal branches that derive from anterior segmental arteries, which run along the vertebral bodies from anterior to posterior, forming the anterior spinal artery which then forms a complex vascular plexus (periosteal and metaphyseal arteries) that irrigates the vertebral bodies and their end plates. The epidural region has, as well, a large anastomotic venous system known as Batson's plexus. The intervertebral discs are avascular and sterile structures, only the outer annulus fibrosus has a few blood vessels that remain from the embryonic development, explaining their incompetent response toward an infection. The infectious process can spread through the blood vessels, the epidural space, or the paraspinal soft tissue, potentially leading to contiguous vertebral and disc involvement or an abscess formation and spinal cord compression [3].

Spondylodiscitis in the adult commonly has an insidious onset, with nonspecific signs of systemic infection, such as fever, chills, and weight loss. Typical early symptoms include localized neck and back pain that worsens with activity, due to irritation of the paraspinal musculature and the sensory nerves of the spine. Patients who are chronically ill, particularly diabetics, are susceptible to present with a more severe clinical picture such as paraparesis or paraplegia, due to epidural involvement [4, 5].

Laboratory tests are a useful tool to aid diagnosis and response to treatment, blood markers frequently used are C-reactive protein (CRP) and blood sedimentation rate (BSG), which can be elevated in an acute phase of the disease (75%–98%). Other parameters include leukocytosis, which may not be present in all cases and elevated procalcitonin (PCT), but it is more useful in case sepsis is suspected. The most reliable method to diagnose and isolate a pathogen is the biopsy of bone and disc material, with a detection rate of up to 68%–93% [6].

Radiographic imaging is essential to diagnose and determine the grade of affection, being X-rays the first-line study, which may show intervertebral disc height loss, endplate erosion, and annular calcification of the affected segment. It is useful to assess disease progression and deformities but only present detectable alterations after a couple of weeks from the onset of the infection. Computed tomography (CT) may be used in conjunction with intravenous contrast or myelography to detect discitis in an early stage, revealing sclerosis and destruction of the intervertebral disc, endplates, and/or vertebral bodies, and inflammation of adjacent paraspinal soft tissue. Magnetic resonance imaging (MRI) remains the gold standard, and it has a high specificity and sensitivity at 96% and 92%, respectively, being able to distinguish between spondylodiscitis, neoplasia, or degeneration. Other modalities such as positron emission tomography (PET-CT) and multiphase bone scintigraphy with technetium begin to have an important role in diagnosis [7].

The treatment of bacterial spondylodiscitis aims to eliminate the infectious process, stabilize pathological instability, restore balance, and preserve neurological function. Most guidelines recommend 6 weeks of intravenous or oral antibiotic-targeted therapy, but there is still no high evidence-level consensus [8].

Prolonged antibiotic therapy and early surgical interventions play an important role in the reduction of morbidity and mortality. Main surgical criteria are sepsis, neurologic deficit, evidence of an abscess, spinal instability, and deformity. Procedures such as I&D, decompression, and instrumentation are often critical to treat the focus of infection, stop neurologic deterioration, and allow for a faster recovery and mobilization of the patient [9].

Adult spontaneous spondylodiscitis is less common than in children, leading to a delay in diagnosis and a higher morbimortality. Published literature has mainly focused on diagnostic imaging techniques from a nonorthopedic point of view; therefore, we believe this case exhibits the importance of an early and aggressive antibiotic and surgical treatment to improve patient outcomes.

## 2. Clinical Case

A 39-year-old female patient presented to external consult after presenting on May 2021, a fall from her height secondary to a syncopal episode, as well as anomic aphasia, dizziness, and short-term memory loss. She presented again on August 2021, after another syncopal episode, as well as the gradual loss of sensibility and strength in all 4 extremities, and intense pain located in the cervical and thoracic regions.

During the physical examination, the patient remained alert, orientated, and cooperative, with oxygen saturation of 88%, a rhythmic heartbeat, globally diminished sensibility (1/2 ASIA), and strength (3/5 ASIA) in the upper extremities. A 3 cm diameter flyctena was documented on the medial aspect of the left heel, as well as spontaneous and involuntary movements of the left leg. A patient with a history of urinary tract infection was treated with ciprofloxacin for three weeks. Laboratories were obtained, showing chronic anemia grade 2 (WHO), platelets 515 K/*μ*L and leucocytes 6 K/*μ*L.

**Figure 1:**
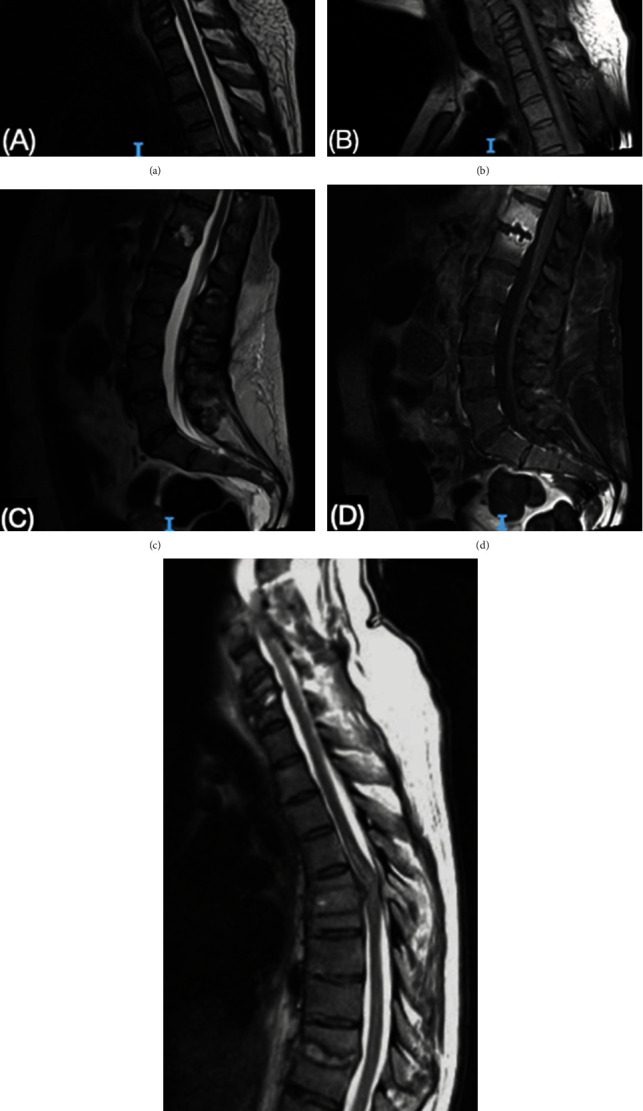
MRI showing the cervical, thoracic, and lumbar spine. (a, b) At the cervical spine, loss of the lordotic angle is demonstrated; destruction of the 6th cervical vertebrae is noted. In C3-C4, there is a collapse of the disc with changes in the signal intensity of the vertebral endplates, which are hyperintense in STIR T2 and isointense in T1 as well as an epidural abscess of 27 × 4.5 mm and encompasses the entire topography of C3 and C4, displacing and compressing the spinal cord. (c, d) At the level of the T12 and L1 vertebral bodies, respectively, in their lower and upper endplates, there is an erosive lesion of 17 × 18 mm involving the intervertebral disc, hyperintense on T2, and hypointense on T1, presenting wall enhancement following contrast. (e) The morphology and height of vertebral bodies T6 and T7 presented morphology alterations due to the destruction of the lower and upper vertebral endplates, respectively, associated with an expansive mass including the intervertebral disc, noted as hypointense on T1 and enhanced on T1 FatSat with gadolinium, and these changes are also observed in the epidural space, surrounding the spinal cord.

A series of X-rays and MRI were performed (Figure 1), which demonstrated multiple erosive and expansive lesions in the intervertebral discs and endplates in all segments of the spine, the most important being at the cervical region (C3-C7), with destruction and collapse of C6 and an abscess compressing the spinal cord. At the thoracic region (T6-T7), there was noted another abscess with significant compression of the spinal cord as a well as endplate and disc destruction, and at the thoracolumbar junction (T12-L1), an early process of spondylodiscitis was noted, with the erosion of the disc and endplates and an abscess in the medullary canal.

On November 2021, the patient was admitted for surgery under the diagnosis of spondylodiscitis to stop the progression of the severe neurological deficit. A staged surgical approach was preferred over a single surgical approach for two main reasons: first, the patient presented with suboptimal health status and second, it was the surgical team's choice of approach.

**Figure 2:**
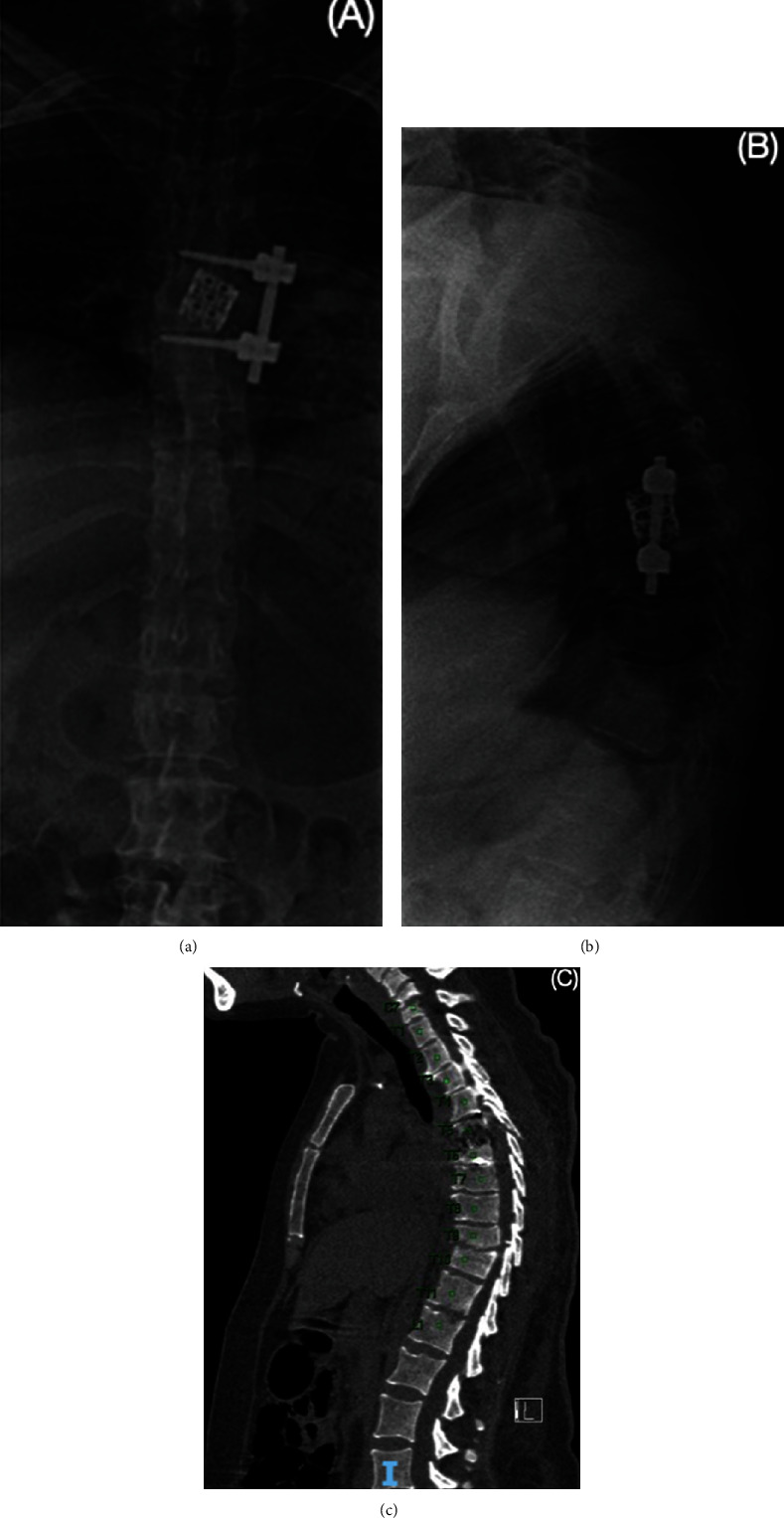
Postoperative x-ray of the thoracic spine: (a, b) postoperative changes are visualized at the level of the bony structures by left lateral instrumentation, with an intersomatic cage prosthesis at the level of T6-T7; (c) bone structures with postsurgical changes due to instrumentation in the thoracic spine, observing fixation screws and bar from the body of T4 to the body of T7.

Based on physical exploration and neurological deficit, the first surgery was conducted in the most symptomatic region, the thoracic spine. A lateral retropleural thoracic approach was conducted, an osteotomy of the rib was performed, and selective lung intubation was needed to deflate it during the procedure. Affected levels (T6-T7) were identified and before I&D, multiple samples were obtained for cultures and histopathological study. Debridement included a corpectomy of T6, stability of the spine was achieved by placement of a titanium cage filled with allograft and matrix, and fixation was done with lateral T5-T7 screws and rod. A chest tube was placed and maintained for the next 72 hours, and broad-spectrum antibiotic therapy was initiated with vancomycin and meropenem awaiting culture results (Figure 2).

**Figure 3:**
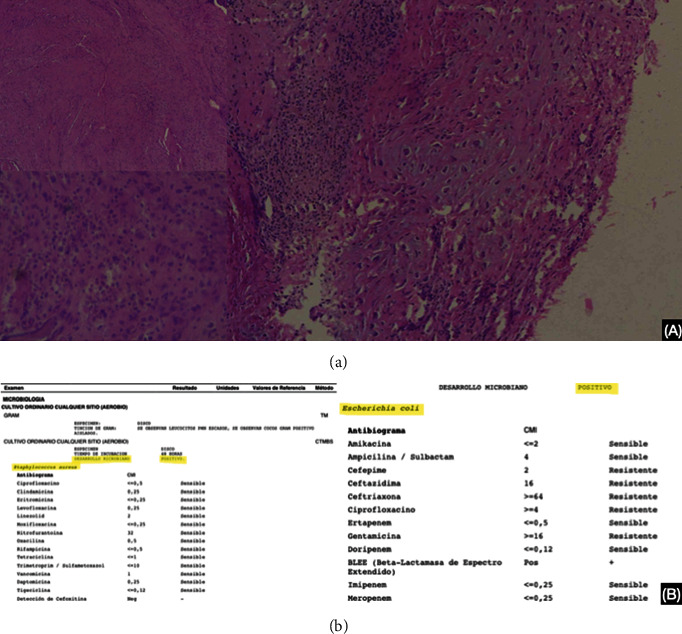
(a) Pathology report of T6-T7 intervertebral disc shows extensive fibrosis with mixed inflammatory infiltration, bone and cartilage fragments, and reactive-looking atypia. There was no evidence of granulomas. (b) Cultures isolated *Escherichia coli* extended-spectrum beta-lactamases (ESBL) and Gram-positive cocci *Staphylococcus aureus*.

The biopsy sample demonstrated extensive fibrosis with a mixed inflammatory infiltrate and fragments of bone and cartilage with reactive-looking atypia. Under PAS and Ziehl-Neelsen stains, no microorganisms were identified. The cultures isolated *Escherichia coli* extended-spectrum beta-lactamases (ESBL) and Gram-positive cocci *Staphylococcus aureus*. Rose bengal and GeneXpert MTB/RIF (mycobacterium tuberculosis/rifampin) studies were negative for tuberculosis and brucellosis (Figure 3). The patient was discharged home after clinical improvement of pain and function, and infectology indicated a 6-week oral antibiotic course at home with ertapenem, trimethoprim/sulfamethoxazole, and doxycycline.

**Figure 4:**
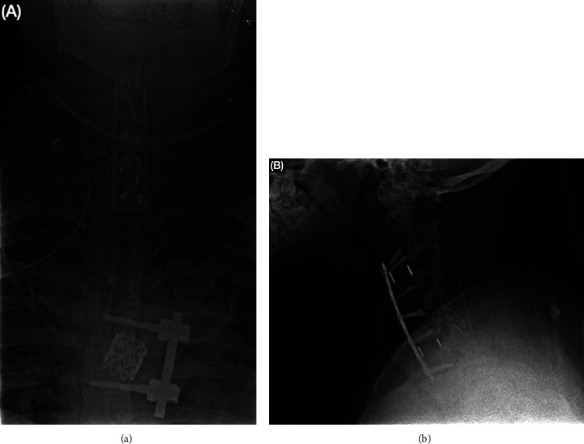
Postoperative x-ray of the cervical spine: (a) surgical material consisting of a 64 mm titanium plate and transcortical locked screws placed from C3 to C7; (b) intersomatic cages at C3-C4 and C6-C7 levels. The amplitude of the intervertebral spaces C2-C3, C4-C5, and C5-C6 is reduced.

A second surgical intervention was scheduled on January 2022 after the patient presented with deterioration of sensibility and strength in the upper extremities. The cervical spine X-rays showed progression of the collapse and deformity noted in the previous MRI. An anterior cervical approach (Smith-Robinson) on the right side was performed, I&D of the prevertebral and medullary canal was carried out and multilevel discectomies were done in levels C3-C4 and C6-C7 and replaced with PEEK cages with matrix, and fusion and stabilization were maintained with an anterior 64 mm titanium plate and locked screws (Figure 4). After the surgical procedure, the patient reported minimal neck pain but right leg pain persisted, mobilization outside of bed with assistance was allowed, and the patient was discharged home with antibiotic and analgesic treatment with neurological rehabilitation for 3 months.

**Figure 5:**
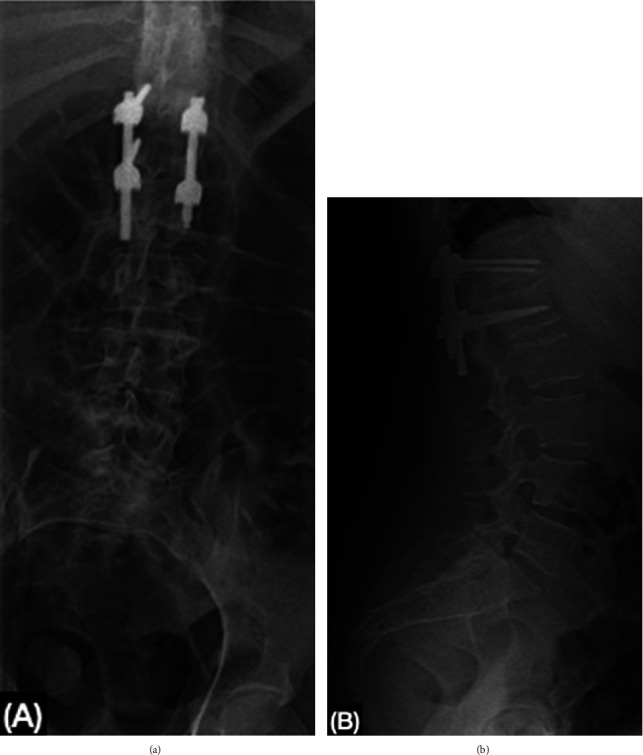
Postoperative x-ray of the lumbar spine: (a) transpedicular screws and rod are seen between T12 and L1. The form and height of vertebral bodies are preserved; (b) appropriate amplitude of inter somatic space. A lumbar angle of 57° indicates physiological lordosis.

In April 2022, during follow-up, low back pain while walking and intractable pain in the right leg continued despite rehabilitation and analgesic medication. The patient was readmitted and scheduled for surgery, a direct posterior thoracolumbar approach was carried out at T12-L1 for I&D, discectomy, foraminal decompression, and fixation with transpedicular screws and rods. Evolution at 24 and 36 hours post-op showed pain decline and increased strength in the lower extremities (Figure 5). The patient was discharged home to continue with rehabilitation therapy in an ambulatory center.

## 3. Results

Multiple surgical procedures were performed at 3 different spinal segments within 5 months, including discectomies, a corpectomy, and multiple arthrodeses. Postoperative follow-up was carried out through external consultation, laboratory tests, and imaging studies, and neurological rehabilitation therapy was carried out between procedures and in an early manner. The patient presented adequate post-op evolution, achieving resolution of pain and recovery of strength in the upper and lower extremities.

## 4. Discussion

Spondylodiscitis has a growing incidence due to an increment of the elderly and chronically ill population. It is estimated that it accounts for 2-7% of all osteomyelitis, with an incidence of 1/100,000 to 1/250,000 cases per year. However, in less developed countries such as Mexico and most of Hispanic America, compared to worldwide statistics, a higher prevalence of up to 11% has been reported in patients attended for chronic low back pain, due to a higher prevalence of comorbidities such as diabetes, chronic alcoholism, collagen vascular disease, liver cirrhosis, end-stage renal failure, steroid use, and human immunodeficiency. At least one of these linked comorbidities will be present in 45-79% of patients with spondylodiscitis, being diabetes the most common (11-31%). Neurological impairment may present in up to 30% of cases, caused by radiculopathy or myelopathy. As for neurologic manifestations, the cervical spine has the highest prevalence, followed by the thoracic spine and lastly, the lumbar spine. The primary focus of infection should try to be identified, the urinary tract, the most frequent location, accounting for 30.8% of all cases. Uropathogenic organisms such as *E. coli* and Proteus species are the most common, and other locations such as a vascular access site should be investigated (7.7%), where *Pseudomonas aeruginosa*, *Klebsiella* spp, and other Gram-negative organisms can be found. Less frequent causes are immunodeficiency (6.4%), intestinal infection (5.1%), and bacteremia related to endocarditis (5.1%) [1, 10].

## 5. Conclusion

Spondylodiscitis is a rare disease with a rising incidence due to a susceptible population, high suspicion and effective diagnostic tools are necessary to obtain early diagnosis and prevent neurologic deterioration. In this case, the diagnostic suspicion was based on the clinical history of UTI and imaging studies such as an MRI. Unfortunately, our patient took months before seeking medical evaluation, with the high progression of the disease.

In contrast to major joint infections, the use of metallic implants and grafts does not jeopardize the successful treatment of infectious spondylodiscitis as the spine needs to be stabilized to restore balance and deformity and prevent further degeneration. For spinal stabilization, various metal implants can be used, such as in this case. The patient recovered sensibility, strength, and mobility during the 6 months follow-up, being able to walk without pain. Further research needs to assess the role of metallic implants and grafts in infectious diseases of the spine as well as oral and intravenous therapy goals to improve long-term patient outcomes [9, 11].

## Data Availability

The authors confirm that the data supporting the information of this clinical case study were derived from the following resources available in the public domain.
